# Case report: Advances in treating ligyrophobia with third-generation ACT approach

**DOI:** 10.3389/fpsyt.2024.1425872

**Published:** 2024-09-26

**Authors:** Flavia Marino, Germana Doria, Adele LoPresti, Stefania Gismondo, Chiara Failla, Giovanni Pioggia

**Affiliations:** ^1^ Institute for Biomedical Research and Innovation (IRIB), National Research Council of Italy (CNR), Messina, Italy; ^2^ Faculty of Psychology, International Telematic University Uninettuno, Roma, Italy; ^3^ Academy of Cognitive Behavioral Sciences of Calabria ASCoC, Calabria, Italy; ^4^ Classical Linguistic Studies and Education Department, Kore University of Enna, Enna, Italy

**Keywords:** anxiety, acceptance and commitment therapy, ligyrophobia, case report, psychoterapy

## Abstract

**Background and aims:**

Giulia, an 11-year-old girl diagnosed with ligyrophobia, has been experiencing intense anxiety related to loud noises since the age of two. This case report aims to explore the efficacy of Third-Wave Cognitive Behavioral Therapy, specifically Acceptance and Commitment Therapy (ACT), in addressing Giulia’s anxiety and avoidance behaviors. The primary goal is to examine the impact of ACT on reducing ligyrophobic symptoms and enhancing psychological flexibility.

**Methods:**

The therapeutic intervention spanned 24 sessions and was divided into two phases. The initial phase focused on emotional competence using characters from the movie “Inside Out,” aiding Giulia in recognizing and understanding her emotions. The subsequent phase targeted the six processes of psychological inflexibility identified in ACT, fostering increased flexibility.

**Results:**

Throughout the intervention, Giulia demonstrated significant improvements in anxiety symptoms, as evidenced by a decrease in Subjective Units of Distress (SUD) scores. Avoidance behaviors also diminished, and Giulia exhibited enhanced mindfulness skills. She became more cognizant of her emotional experiences and more certain of her personal ideals as a result of the therapy. Giulia’s active participation and commitment to exposure exercises led to a substantial reduction in ligyrophobic reactions.

**Discussion:**

The successful application of ACT in Giulia’s case suggests that targeting psychological inflexibility through mindfulness and values clarification can be effective in treating ligyrophobia in children. The integration of metaphors and creative exercises proved valuable in engaging Giulia and fostering therapeutic progress. The findings underscore the importance of a tailored, third-wave therapeutic approach in addressing specific phobias in pediatric populations.

## Introduction

A specific phobia is characterized by an intense and persistent fear of a particular situation or object, accompanied by symptoms of anxiety, distress, and a tendency to avoid the feared stimulus (1). These phobias can significantly impact daily functioning (2). Moreover, they have been linked to a diminished quality of life (3, 4), and are indicative of potential future mental health issues (5, 6). Ligyrophobia, also referred to as sonophobia or phonophobia, is a specific phobia characterized by an excessive and irrational fear or aversion to loud noises. Individuals afflicted by ligyrophobia may experience immediate reactions ranging from heightened anxiety to panic when exposed to loud sounds. In an effort to cope with this fear, individuals may go to great lengths to organize their lives in a manner that minimizes their exposure to loud noises. While anyone can find a loud noise surprising or annoying, a person with a specific phobia of loud noises has more extreme symptoms, from anxiety to panic. The reaction can occur during the noise, before it happens, or after the noise has stopped. Symptoms vary in severity for each person and may include (1) fear, shortness of breath, increased heart rate, sweating, dizziness, anxiety, screaming, fainting, chest pain, in fact, as Schröder A et al. (2013) (7) suggest misophonia cannot be specifically classified among the current DSM-5 disorders. Furthermore, as highlighted by the research of Hadjipavlou G. et al., 2008 (8) the cause of selective sound intolerance remains unclear. A possible explanation is found in the audiology and otolaryngology literature, where the term ‘misophonia’ is used to describe a condition characterized by reduced sound tolerance (9). A person with a phobia of loud noises often takes steps to avoid loud noises in daily life. Both symptoms and avoidance can create stress and interfere with your ability to carry out daily activities. In the literature (10), we can find different descriptions of sound tolerance conditions for example Phonophobia or ligyrophobia is defined as an anticipatory fear of sound in which people experience anxiety and avoid settings where they expect certain sounds to increase comorbid conditions such as tinnitus or cause discomfort or pain. This distinguishes phonophobia from hyperacusis, a condition in which physical discomfort or suffering is caused by sound levels that are ordinarily tolerated by others. It also varies from misophonia, which causes extreme emotional reactions to certain sounds, such as chewing or sniffing, regardless of their volume (7). Noise sensitivity, on the other hand, refers to a heightened reactivity to sounds, which frequently results in overall discomfort or feeling overwhelmed in noisy surroundings, regardless of the sound’s loudness. The specific cause of ligyrophobia is unknown. In general, there are different reasons why people develop phobias. They may be due to a learned behavior (such as observing a parent who has the phobia (11), genetics (12), or an experience that led to the development of the fear or phobia (13). According to research on childhood anxiety disorders (CADs) (14, 15), cognitive behavioral therapy (CBT) is the most researched intervention. It is also often suggested as the first line of treatment for CADs (16). The evidence that is currently available in favor of CBT’s use includes results showing that it works better than placebo and waitlist control, is comparable to medicine, and outperforms sham therapy controls (supportive environments devoid of active therapeutic elements (15, 17) Additionally, CBT has been applied as a reference point to prove outcome equivalency (18). ACT is a third-wave psychotherapy aimed at improving psychological flexibility, or the ability to be fully aware, open to one’s experience and to act on the basis of one’s values not only by trying to avoid disturbing feelings, thoughts, memories or desires but by increasing one’s willingness to act despite the presence of unpleasant sensations (12).

## Methods

During the assessment phase, two primary instruments were utilized to evaluate Giulia’s anxiety levels: the Multi-dimensional Anxiety Scale for Children (MASC) (19) and the State-Trait Anxiety Inventory (STAI-Y) (20). The MASC is designed to assess anxiety levels, where elevated scores indicate high anxiety (T0 = 66), similar to the STAI-Y(T0 = 61). Additionally, the Italian-Child and Adolescent Mindfulness Measures (I-CAMM) (21) and the Italian-Avoidance and Fusion Questionnaire - Youth (I-AFQ-Y8) (22) were employed to assess psychological flexibility and inflexibility. The I-CAMM measures the ability to be in the present moment, with higher scores indicating a tendency to lack this ability. The I-AFQ-Y8 assesses the tendency to get caught up in one’s thoughts, where higher scores indicate psychological inflexibility in this process. Giulia’s scores demonstrated good mindfulness skills with a score of 37 on the I-CAMM and psychological inflexibility with a score of 30 on the I-AFQ-Y8. The treatment plan using the third-generation ACT intervention lasted for a total of 24 weeks, divided into four parts. The first phase (Phase 0) was for the initial assessment and included two sessions. In Phase 1, which consisted of five sessions, we focused on helping with emotional understanding and skills. The longest phase was Phase 2, with 13 sessions. Here, we worked on specific aspects related to the six core components of the ACT approach: acceptance, defusion, values, commitment action,self as context, present moment (13). Phase 3 assessed how well the treatment worked after completion, and Phase 4 was a follow-up to see if the positive effects lasted over time. This structured approach helped thoroughly explore the effectiveness of the intervention in improving psychological flexibility and addressing ligyrophobia in children.

### Treatment format

### Design of the case report

The case study was conducted to analyze and illustrate an individual treatment path regarding the effectiveness of a third-generation cognitive behavioral intervention applied to a little girl. An exemplary reduction in symptoms and a young girl’s satisfaction with the success of the treatment were described.

## Case study

### Case history

Giulia, an 11-year-old girl, faces anxiety issues linked to a fear of loud noises since the age of two. Her anxiety specifically arises in social settings involving items like balloons, champagne bottles, and fireworks. The concern is that these fears might hinder Giulia’s social participation. During discussions about the issue, the mother expresses worry rapidly, while the father minimizes it, attributing responsibility to the mother’s excessive anxiety. Giulia’s daily routine involves managing homework and private lessons, spending time with maternal grandparents. Despite a previous interest in dance, she abandoned it due to academic pressures. Her father, managing a newsstand, wakes up early and often feels fatigued. The mother, working in real estate, juggles multiple responsibilities and cares for her ailing father-in-law. Additionally, she harbors an irrational fear of doves, the origin of which is unknown. At 18 months, Giulia developed a strong fear of loud noises during a religious festival. This fear extends to crowded places and sounds resembling fireworks. The mother is troubled by Giulia’s avoidance behaviors at events involving balloons and her intense reactions to fear-associated sounds. A school incident highlighted Giulia’s struggle in handling fear. An appointment for treatment has been arranged, with hopes for parental involvement, though the father is unable to participate due to work commitments.

### Diagnostic path

Giulia, exhibits symptoms consistent with Specific Phobia Disorder, Other Type, according to the DSM-5. She displays an intense and persistent fear of objects generating loud noises, expressed through crying and avoidance behaviors. This leads to significant social and relational distress, hindering her participation in social events. Her anxiety is specifically linked to situations involving loud noises, excluding other medical conditions or mental disorders. The diagnostic classification suggests Ligyrophobia (300.29 F40.298). The diagnostic process undertaken allowed us to carry out an accurate differential diagnosis by evaluating other mental health conditions that can also present with a significant fear of loud noises. One such condition is autism spectrum disorder (ASD). Individuals with ASD exhibit heightened sensory sensitivity, including an aversion to loud noises. Unlike Specific Phobia Disorder, ASD is characterized by additional core characteristics such as difficulties in communication and social interaction, as well as limited and repetitive patterns of behavior, interests or activities. In Giulia’s case, her symptoms are linked exclusively to the fear of loud noises without the presence of social communication deficits or restricted/repetitive behaviors, ligyrophobia therefore remains the most accurate diagnosis. Another condition considered in the differential diagnosis includes panic disorder, which although involves episodes of intense fear and physical symptoms, typically includes a broader range of triggers and is characterized by unexpected panic attacks.

### Description of the treatment

**Figure 1:**
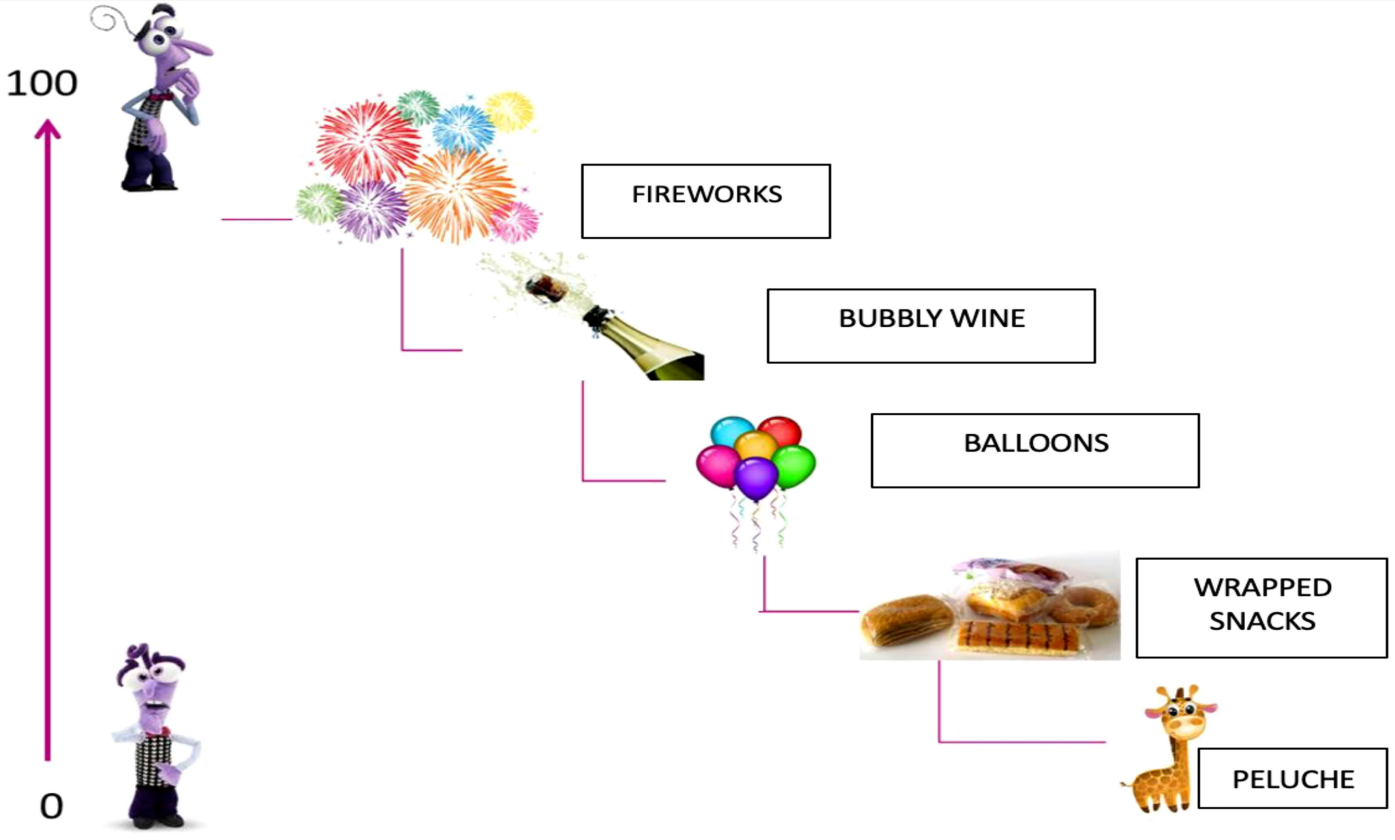
How the SUDS scale was proposed to Giulia.

The treatment for Giulia aims to reduce anxiety manifestations and avoidance behaviors related to phobic objects and anxiety-inducing situations. In 24 sessions (see **Table 1**), Giulia demonstrates good cooperation and motivation. Drawing inspiration from the movie ‘Inside Out,’ the therapeutic process is structured into four phases, actively involving Giulia and her mother. In the zero phase, during the assessment phase, two key instruments, the Multi-dimensional Anxiety Scale for Children (MASC) and the State-Trait Anxiety Inventory- STAI-Y, were employed to evaluate Giulia’s anxiety levels. The MASC revealed elevated scores in physical symptoms, separation, and avoidance, indicating significant anxiety symptoms encompassing both physical manifestations and habitual traits. Additionally, the Italian-Child and Adolescent Mindfulness Measures (I-CAMM) (16) and the Italian-Avoidance and Fusion Questionnaire - Youth - I-AFQ-Y8 (17) were used to assess psychological flexibility and inflexibility. Giulia demonstrated good mindfulness skills with a score of 37 on the I-CAMM and a score of 30 on the I-AFQ-Y8, indicating psychological inflexibility. In the first phase, during the initial treatment phase, the focus was on Giulia’s emotional competence, using characters from the movie ‘Inside Out’ to help her recognize her emotions. Through Joy, Anger, Fear, Sadness, and Disgust, the emphasis was on identifying bodily signals associated with emotions. Giulia learned to distinguish heart sensations from other bodily sensations, contextualizing them in personal experiences. This process assisted Giulia in identifying fear as the root of her distress and understanding the relationship between anxiety and fear. Once Giulia recognized emotions and their physical manifestations, the second phase, which spanned 13 sessions, involved identifying the six processes related to her “psychological inflexibility” and guiding her towards greater flexibility. Initially, self-representation was encouraged through the completion of the ‘Identity Card’ to enhance self-awareness and self-acceptance. Subsequently, the defusion process was addressed, utilizing the metaphor of libraries in Giulia’s mind managed by Fear and Joy. The concept of values was explored through the story of the ‘Magic Lamp’ and the drawing of ‘Her Garden,’ aiding Giulia in identifying what truly matters to her. The phase of committed actions was initiated alongside a gradual exposure process, where Giulia faced phobic objects and anxiety-inducing situations following the SUD scale compiled during the assessment phase. To quantify the anxiety experienced in response to specific stimuli, it was deemed appropriate to use the conventional Subjective Units of Distress Scale (S.U.D.S.). By explaining the purpose of this scale, the participant, Giulia, was asked to establish a clear communication code to express the level of fear associated with a situation or stimulus (see **Figure 1**). She was asked to report the degree of fear on a scale from 0 to 100, where 100 represents the maximum fear (losing control of actions, feeling confused, wanting to escape) and 0 indicates the total absence of anxiety. Using this method, a hierarchy of anxiety-evoking stimuli was created with Giulia, ordered based on the intensity of the anxiety they produced.

**Table 1:** Overview of content of main sessions.

Session	Intervention content – Main sessions	Session
**Phase 0 (t0)**	Assessment	**1/2**
**Phase 1**	Emotional Competence Training	**3-7**
**Phase 2**	Acceptance	**8/9**
	Defusion	**10/11**
	Values	**12/13**
	Commitment Action	**14-16**
	Self as Context	**17/18**
	Present Moment	**19/20**
**Phase 3** **(t1)**	Post-assessment	**21/22**
**Phase 4** **(t2)**	Follow-up	**23/24**

**Figure 2:**
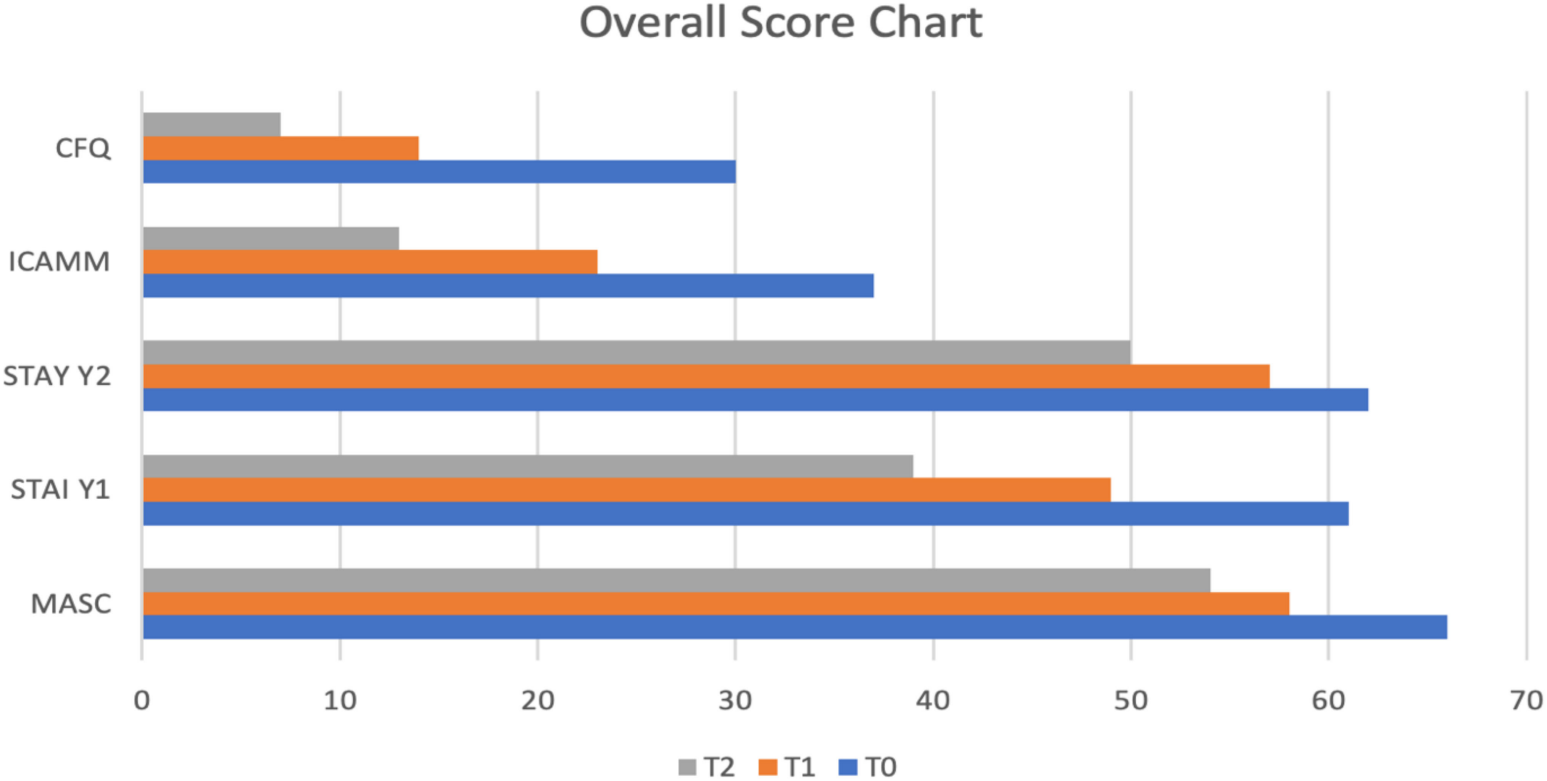
Overall Score Chart.

The treatment, which included Giulia’s mother, involved a gradual exposure to feared stimuli with the therapist’s support. With an increased awareness of processes enabling her to manage fear, Giulia progressively simplified her approach to anxiety-inducing situations. To enhance Giulia’s present-moment awareness and prevent her from getting lost in thoughts, each session begins with mindfulness exercises. Giulia actively participates with enthusiasm, showing interest in repeating the exercises at home between sessions. In the third and fourth phases of the intervention, the focus shifted towards assessing the outcomes using key instruments that measured Giulia’s anxiety levels. The Multi-dimensional Anxiety Scale for Children (MASC) and the State-Trait Anxiety Inventory- STAI-Y were re-administered during these phases. Notably, the results reflected a positive trend of Giulia’s progress. In the third phase, there was a discernible decrease in both trait anxiety symptoms, indicated by a score of 57, showcasing an improvement in her overall anxiety profile. Moreover, the temporary disruption of the emotional continuum (state anxiety) decreased significantly to a score of 49. Moving into the fourth phase and the follow-up assessments, the positive trajectory continued, with a gradual decrease in scores related to avoidance and cognitive fusion. The findings suggest a sustained improvement in Giulia’s ability to manage and cope with anxiety-inducing situations over time. Additionally, in the evaluation of mindfulness skills, there was a notable increase in awareness of the experience, underscoring the effectiveness of the intervention in enhancing Giulia’s mindfulness and emotional regulation abilities. The outcomes from the third and fourth phases collectively highlight the therapeutic impact on Giulia’s anxiety symptoms, emphasizing the enduring positive changes observed in both behavioral and psychological dimensions (see **Table 2**, **Figure 2**).

**Table 2:** Overview of sum scores of measured comorbidities at T0, T1 and T2.

Construct	Questionnaire	Sum scores		
		T0	T1	T2
**Anxiety**	Multi-dimensional Anxiety Scale for Children	66	58	54
**Anxiety**	State-Trait Anxiety Inventory Y1	61	49	39
**Anxiety**	State-Trait Anxiety Inventory Y2	62	57	50
**Mindfulness**	Italian-Child and Adolescent Mindfulness Measures	37	23	13
**Cognitive Fusion**	Italian-Avoidance and Fusion Questionnaire - Youth	30	14	7

## Discussion

Psychotherapy is the cornerstone of treatment for specific phobias, like ligyrophobia, or fear of loud noises (23, 24). Among the numerous therapeutic techniques, exposure therapies and CBT have demonstrated significant success. Exposure treatment is a key component of cognitive behavioral therapy, and it is especially successful in treating specific phobias. The goal is to reduce the fear response through habituation and desensitization. CBT integrates exposure treatment and cognitive restructuring approaches. This comprehensive approach focuses on the maladaptive cognitive patterns that underpin the phobia. Third-wave therapies have recently emerged as viable treatments for a wide range of psychiatric problems, including specific phobias (25). Exposure treatment in ACT and classical CBT shares similarities but also notable differences. ACT integrates exposure treatment within a broader framework of psychological flexibility. The goal is not necessarily to lessen the fear reaction, but rather to alter the patient’s connection with their fear. ACT stresses acceptance and mindfulness, urging patients to feel their fear without judgment and to commit to acts that are consistent with their values, even in the face of fear. The purpose of this case report study was to demonstrate the effectiveness of ACT in treating ligyrophobia. The successful application of ACT in Giulia’s case provides valuable insights into the treatment of ligyrophobia in children by targeting psychological inflexibility through mindfulness and values clarification. The integration of metaphors and creative exercises emerged as a particularly effective strategy, actively engaging Giulia and fostering therapeutic progress. Examining the results of the anxiety assessments, Giulia displayed elevated scores in areas associated with ligyrophobia, such as physical symptoms, separation, and avoidance. The STAI-Y highlighted high levels of both state and trait anxiety, indicating the significant impact of ligyrophobia on her emotional well-being. The improvements in Giulia’s scores on the Italian-Child and Adolescent Mindfulness Measures (I-CAMM) are strongly indicative of her progress in therapy. This aligns with the broader therapeutic goal of promoting mindfulness as a coping mechanism for anxiety symptoms. Similarly, the Italian-Avoidance and Fusion Questionnaire - Youth - I-AFQ-Y8 underscored Giulia’s progress in terms of psychological inflexibility. The decrease in scores over the course of the treatment phases reflects a positive trend toward greater psychological flexibility, indicating a shift toward healthier coping mechanisms. These collective findings highlight the effectiveness of a tailored, third-wave therapeutic approach, such as ACT, in addressing specific phobias in pediatric populations. The emphasis on mindfulness and values clarification, coupled with creative interventions, proves instrumental in not only reducing anxiety symptoms but also promoting a more flexible and adaptive psychological response to fear-inducing stimuli. It is crucial to acknowledge the individualized nature of these interventions, considering Giulia’s unique presentation and experiences. The successful outcomes further emphasize the importance of adapting therapeutic techniques to suit the developmental and emotional needs of children, paving the way for more targeted and impactful interventions in pediatric anxiety disorders.

## Conclusion

This case report provides compelling evidence for the favorable outcomes achieved through the application of ACT in the treatment of ligyrophobia in an 11-year-old girl named Giulia. The intervention not only successfully alleviated anxiety symptoms but also contributed to a significant reduction in avoidance behaviors. Giulia’s enhanced capacity for mindfulness and a shift towards values-driven actions further underscore the comprehensive impact of ACT on her psychological well-being. The sustained improvements observed in Giulia post-treatment suggest the enduring efficacy of ACT in promoting psychological flexibility among children struggling with specific phobias. The positive changes in her behavior and emotional responses indicate that ACT effectively facilitated adaptive coping mechanisms, allowing Giulia to confront and manage her fears more effectively. The implications of these findings extend beyond the individual case, shedding light on the potential applicability of ACT as a valuable therapeutic approach for pediatric specific phobias, such as ligyrophobia. The intervention’s success in enhancing Giulia’s psychological flexibility emphasizes the importance of addressing not only symptom reduction but also fostering resilience and adaptive functioning in the face of fear-inducing stimuli. This case report adds to the growing body of literature supporting the efficacy of ACT in pediatric populations, suggesting that the principles of acceptance, mindfulness, and value-driven actions can be powerful tools in the therapeutic arsenal for managing childhood phobias. Further research and exploration of ACT in diverse clinical contexts may provide valuable insights into its broader utility and refine its application in pediatric mental health interventions.

## Data Availability

The raw data supporting the conclusions of this article will be made available by the authors, without undue reservation.
